# Targeted sequencing reveals complex, phenotype-correlated genotypes in cystic fibrosis

**DOI:** 10.1186/s12920-018-0328-z

**Published:** 2018-02-13

**Authors:** Maxim Ivanov, Alina Matsvay, Olga Glazova, Stanislav Krasovskiy, Mariya Usacheva, Elena Amelina, Aleksandr Chernyak, Mikhail Ivanov, Sergey Musienko, Timofey Prodanov, Sergey Kovalenko, Ancha Baranova, Kamil Khafizov

**Affiliations:** 1 Moscow Institute of Physics and Technology, Department of Biological and Medical Physics, Dolgoprudny, Moscow Region Russian Federation 141700; 2 Atlas Biomed Group, Moscow, Russian Federation 121069; 3 Central Research Scientific Institute of Epidemiology, Moscow, Russian Federation 111123; 4 Federal Pulmonology Research Institute, Moscow, Russian Federation 105077; 5 Department of Mathematics and Information Technology, St. Petersburg Academic University, St. Petersburg, Russian Federation 195251; 6 The Institute of Molecular Biology and Biophysics, Novosibirsk, Russian Federation 630117; 7 Novosibirsk State University, Novosibirsk, Russian Federation 630090; 8 Research Centre for Medical Genetics, Moscow, Russia; 9 Center for the Study of Chronic Metabolic Diseases, School of Systems Biology, George Mason University, Fairfax, VA USA

**Keywords:** *NGS* next generation sequencing, *CFTR* cystic fibrosis

## Abstract

**Background:**

Cystic fibrosis (CF) is one of the most common life-threatening genetic disorders. Around 2000 variants in the CFTR gene have been identified, with some proportion known to be pathogenic and 300 disease-causing mutations have been characterized in detail by CFTR2 database, which complicates its analysis with conventional methods.

**Methods:**

We conducted next-generation sequencing (NGS) in a cohort of 89 adult patients negative for p.Phe508del homozygosity. Complete clinical and demographic information were available for 84 patients.

**Results:**

By combining MLPA with NGS, we identified disease-causing alleles in all the CF patients. Importantly, in 10% of cases, standard bioinformatics pipelines were inefficient in identifying causative mutations. Class IV-V mutations were observed in 38 (45%) cases, predominantly ones with pancreatic sufficient CF disease; rest of the patients had Class I-III mutations. Diabetes was seen only in patients homozygous for class I-III mutations. We found that 12% of the patients were heterozygous for more than two pathogenic CFTR mutations. Two patients were observed with p.[Arg1070Gln, Ser466*] complex allele which was associated with milder pulmonary obstructions (FVC 107 and 109% versus 67%, CI 95%: 63-72%; FEV 90 and 111% versus 47%, CI 95%: 37-48%). For the first time p.[Phe508del, Leu467Phe] complex allele was reported, observed in four patients (5%).

**Conclusion:**

NGS can be a more information-gaining technology compared to standard methods. Combined with its equivalent diagnostic performance, it can therefore be implemented in the clinical practice, although careful validation is still required.

**Electronic supplementary material:**

The online version of this article (10.1186/s12920-018-0328-z) contains supplementary material, which is available to authorized users.

## Background

Cystic fibrosis (CF) is one of the most common life-threatening genetic disorders affecting Caucasians, with an approximate frequency of 1 per 3000 people [1, 2] and a carrier frequency of 1 per 20-80 people [3–5], which varies in populations of study [4, 6–8]. In Russian Federation, CF incidence in newborns has been estimated at 1:12,300 to 1:5465 [9–12].

CF is inherited as an autosomal recessive trait and is caused by mutations in the cystic fibrosis transmembrane conductance regulator gene *CFTR*. This gene encodes a large protein consisting of 1480 amino acids and acting as cAMP-dependent chloride channel [13, 14]. The defective protein impairs the water movement across epithelia, which leads to the formation of viscous mucus obstructing the airways of the lungs and ducts of the pancreas [15].

At least 280 different CF-causing mutations have been reported to date. The deletion of the phenylalanine 508 (p.Phe508del or c.1521_1523delCTT) is by far the most common mutation in the Caucasian population, affecting more than two-thirds of all CF patients [7, 16]. Most of other mutations are rare and population-specific. For example, the p.Trp1282* is the most prevalent mutation in the Jewish population [17], with a frequency of 60%, while the p.Glu92Lys (c.274G > A) variant is the primary cause of CF in Chuvashes [18]. Other populations display high allelic heterogeneity [19], including single nucleotide variants (SNVs), short insertions and deletions (indels), and large structural variants. The knowledge of the spectrum and the frequencies of CF mutations in a specific population is a prerequisite for setting up adequate and cost-effective molecular diagnostics.

To date, the detection of *CFTR* mutations in a given patient relies on specific tests developed for common mutations. The detection rate of such mutation panels varies significantly depending on the mutations included and the molecular heterogeneity of each population. As a consequence, some patients with common *CFTR* mutations are immediately diagnosed and do not require any additional study, while for others complete molecular screening of the 27 exons and the regulatory regions of *CFTR* remains a necessity. Numerous multiple-step strategies of *CFTR* were developed. These strategies employ multiplexed quantitative polymerase chain reaction, Sanger sequencing, and multiplex ligation-dependent probe amplification; these combinations remain costly, time-consuming, and labor-intensive [20]. In the meantime, massive parallel sequencing has revolutionized molecular diagnostics for other diseases, allowing the analysis of multiple whole genes concurrently [21]. Recent publications showed the value of this technology for CF diagnostics, though thorough validation is still required due to relatively large deletions, copy number variation and the presence of homopolymer sequences which complicate the sequencing analysis and may influence the diagnostics [22, 23].

Importantly, molecular analysis has been shown to be applicable for determining patient prognosis, thus influencing patient management. Significant effort has been undertaken to understand the genotype-phenotype correlation in CF, including the influence of a combination of disease-causing and modifying *CFTR* mutations, as well as inherited variants of other genes. Moreover, *CFTR* variants inherited in *cis* have been identified [24–28]. Interestingly, some mutations seem to alleviate the underlying CF phenotype [29, 30], while the others tend to worsen prognosis [24]. Detailed studies of complex genotypes may clarify mechanistic underpinnings of the symptoms. In the meanwhile, mutation screening with conventional diagnostic methods may miss some *cis*-positioned mutations, which may obscure evaluation of the patient. Hence, there is a need to analyze the whole *CFTR* sequence, which prompts reevaluation of the established screening strategies in favor of NGS.

Here we assessed the efficacy of targeted re-sequencing for the molecular diagnosis of CF in a panel of 89 patients prescreened for the 30 most frequent CF-causing mutations. The purpose of prescreening was to exclude the most frequent genotype, p.[Phe508del];[Phe508del], thus, facilitating the study of less frequent mutations in a cost-efficient way.

## Methods

### Patient and sample collection

The study cohort comprised a total of 89 unrelated Russian cystic fibrosis patients over the age of 18 years. Diagnosis was confirmed by analysis of clinical presentation and Gibson-Cooke sweat test, with chloride ion concentrations of 60 mmol/L or higher defining positive result. Age at diagnosis was not available for the collection. All subjects were prescreened for the 30 most frequent *CFTR* mutations. For all patients, peripheral blood was collected into EDTA vacutainer tubes (BD).

In Russian cystic fibrosis patients, the frequency of the p.[Phe508del];[Phe508del] genotype is at 33% [31]. In this study, patients with p.Phe508del homozygous mutation were excluded, unless there were indications that the course of their disease may be modified by additional genetic changes. As a consequence, only six patients (7%) harbored p.Phe508del in homozygote were included. Participation in this project was based on the informed consent. All further analyses were based on the archival data that were stored in the database with no current connection to the patients’ identifiers. Genotype-phenotype analysis was performed for 84 patients with informed consents explicitly covering this type of research (94%).

Clinical and demographic characteristics of patients are presented in Table 1. Patients without ultrasound-confirmed morphologic changes in the pancreas and normal levels of fecal elastase-1 were diagnosed with the pancreatic sufficient CF, while all the rest were diagnosed with pancreatic insufficient CF disease.

**Table 1:** Clinical and Demographic characteristics of patients

Basic patient characteristics	
Number of Patients	84
Gender	
Male	40 (48%)
Female	44 (52%)
Age, mean (range)	26 (19-47)
Height, cm mean (range)	168 (142-187)
Weight, kg mean (range)	52 (33-75)
Clinical characteristics	
FVC, % mean (range)	67 (28-120)
FEV, % mean (range)	47 (15-111)
Phenotype	
Mild	38 (45%)
Severe	46 (55%)
Diabetes	
Yes	10 (12%)
No	74 (88%)
Pancreatic sufficiency	
Sufficient	37 (44%)
Insufficient	47 (56%)
Bacterial flora characteristics	
S.aureus (yes - no)	12 - 72
P.aeruginosa (yes - no)	41 - 43
Achromobacter spp. (yes - no)	7 - 77
Stenotrophomonas spp. (yes - no)	1 - 83
E.coli (yes - no)	1 - 83
B.cepacia (yes - no)	22 - 62

### PCR amplification, library construction, and massively parallel sequencing

Genomic DNA was extracted using silica adsorption based Blood DNA isolation kit according to the manufacturer protocol (Biosilica, Novosibirsk, Russia). Enrichment was done by employing a previously designed custom pool of 33 primer pairs. Entire open reading frame and certain intronic regions containing several important disease-causing mutations were covered by amplicons. The total size of the covered region was about 9 kb. Single PCR products were mixed for each patient and purified by silica adsorption; concentrations of PCR products were measured with Qubit® 2.0 Fluorometer (Thermo Fisher Scientific, Inc.) and subjected to library preparation using the NEBNext® Fast DNA Library Prep Set for Ion Torrent™ (New England BioLabs Inc.), according to the protocol. For barcoding of the libraries, the Ion Xpress™ Barcode Adapters Kit was used. Quality assessment of the libraries was carried out on the 2100 Bioanalyzer Instrument, employing the Agilent High Sensitivity DNA Kit. After assessment of library concentrations, equimolar amounts of barcoded libraries were brought to a final concentration of 15 pmol/L. The Ion OneTouch™ system was used to clonally amplify the pooled bar-coded libraries on Ion Sphere™ particles according to the Ion OneTouch™ 200 Template Kit user guide. The subsequently enriched template-positive Ion Sphere particles were loaded onto the Ion 318™ chip and sequenced with the Ion PGM 200 Sequencing Kit (Thermo Fisher Scientific, Inc.) on the Ion Torrent PGM sequencer (two runs with 40 and 49 samples, respectively).

### NGS data analysis

Raw sequence data analysis, including base calling and demultiplexing, was performed using the Torrent Suite Software v.4.0.2 (Thermo Fisher Scientific, Inc.). Reads were preprocessed for a removal of low-quality and too short nucleotide sequences using the Prinseq-lite program [32]. The minimum mean read quality score was set to 25, and required read length was set to 75 base pairs. Approximately 60% of the reads remained after the filtering for each patient using these criteria. The remaining single-end reads were mapped to the GRCh37.p13 human genome employing the Burrows-Wheeler Aligner (BWA-mem, version 0.7.7-r441) [33] with default parameters. After the alignment, primers were trimmed by the in-house scripts. Variant calling was done with the SAMtools package version 0.1.19 [34], with increased per-position maximum read depth (up to 10,000) for SNPs and indels, and employing BAQ recalculation on the fly. Small genetic variations covered by at least 15 reads on both strands and with a minimum mutant allele frequency of 20% were considered for further annotation using CFTR2 (as of February 1, 2015), Universal Mutation Database (UMD) CFTR (as of February 1, 2015), ClinVar (as of February 1, 2015) and HGMD (public version) [35] databases. Variant annotation was performed in accordance with the ACMG guidelines. Mutation class was defined according to consensus of published literature. Common polymorphisms were discarded from the analysis based on population frequency of the mutation (> 5% based on the 1000 Genomes Project) [36]. Recurrent artifact variant calls in the amount of 37, predominantly presented by indels, were discarded from the analysis by employing in-house software. Protein variation annotation was performed using ANNOVAR [37]. The *CFTR* reference sequences NG_016465.4, and NM_000492.3 along with NP_000483.3 were used for variation annotation on genome, cDNA and protein levels, respectively. Initial sorting out of the homopolymeric stretch errors was done with in-house own scripts. A Tablet tool [38] was used for visualization of the alignments and for sorting out false-positive indels adjacent to homopolymer sites or demonstrating significant strand-bias. Mutations in homopolymer sites were called separately employing in-house script.

### In silico prediction of mutations pathogenicity

Assessment of potentially damaging effects of the novel and known missense SNPs in the *CFTR* gene product was conducted using programs SIFT (version 1.03) [39], PolyPhen-2 (version 2.2.2) [40], PROVEAN (version 1.1) [41], Phd-SNP (version of 18.01.2010) [42], PANTHER (version 1.03) [43] and SNP&GO (web server accessed April 2015) [44] as well as the in-house software OGMET (submitted for publication). In-frame indels were assessed by PROVEAN and DDIG-in (web server accessed April 2015) [45].

### Mutation verification

Mutations detected by NGS were verified by Sanger sequencing using the ABI PRISM BigDye Terminator Cycle Sequencing v.2.0 Ready Reaction kit and ABI PRISM 3730 DNA analyzer (Applied Biosystems). MLPA was used to detect large insertions or deletions in patients with only one or no mutations detected as previously described [46].

## Results

### NGS data analysis

**Fig. 1:**
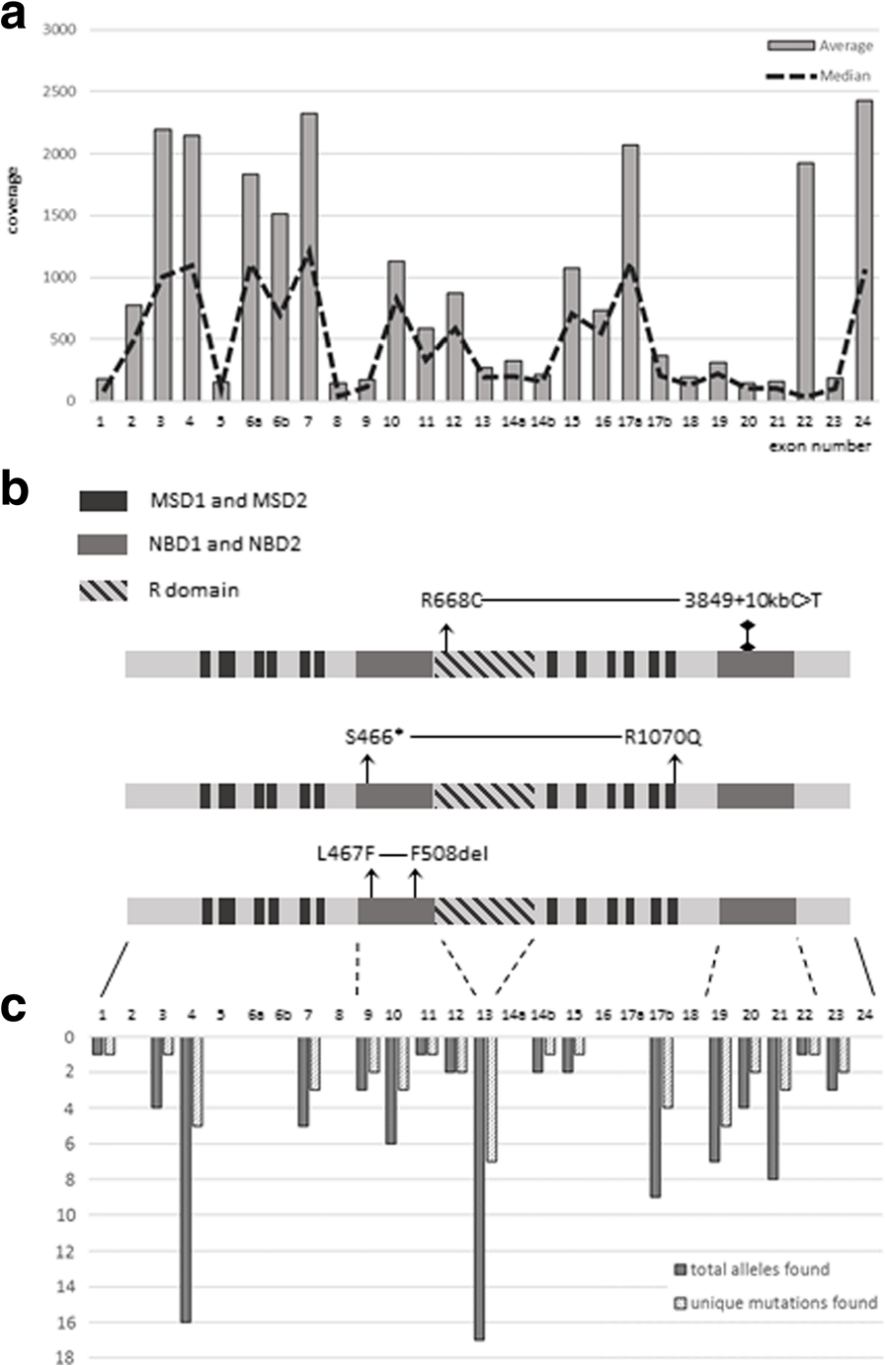
NGS data analysis results. **a**. Mean and median coverage for each *CFTR* exon across 84 samples **b**. Identified complex alleles mapped into *CFTR* gene product. MSD - membrane-spanning domains, NBD - nucleotide-binding domain 1 **c**. Total and unique mutation calls count by *CFTR* exon across 84 samples

In this work, the complete coding sequence of the *CFTR* gene, as well as particular intronic region containing known disease-causing mutation (c.3718-2477C > T), totaling about 9 kb, were covered by 33 amplicons with length varying from 172 up to 328 base pairs. Approximately 96% of all reads were successfully mapped to the reference genome. After preprocessing, read depths of 15× for 87% of the bases and 30× for 84% of the target bases were obtained (Fig. 1a). The overall median coverage was 238×. These results clearly indicate a high-resolution capability of Ion Torrent PGM technology for identifying small genome variations, including point mutations and short indels. On the other hand, due to significant differences in the coverage across samples, which, for some regions, varied up to three orders of magnitude, the large copy number variations could not be detected. For example, the detection failed in patients with known deletion of exons 2 and 3 (p.Ser18_Gly91del) or 6b-10 duplications (c.(743 + 1_744-1)_(1584 + 1_1585-1)dup), common in CF patients [47].

**Fig. 2:**
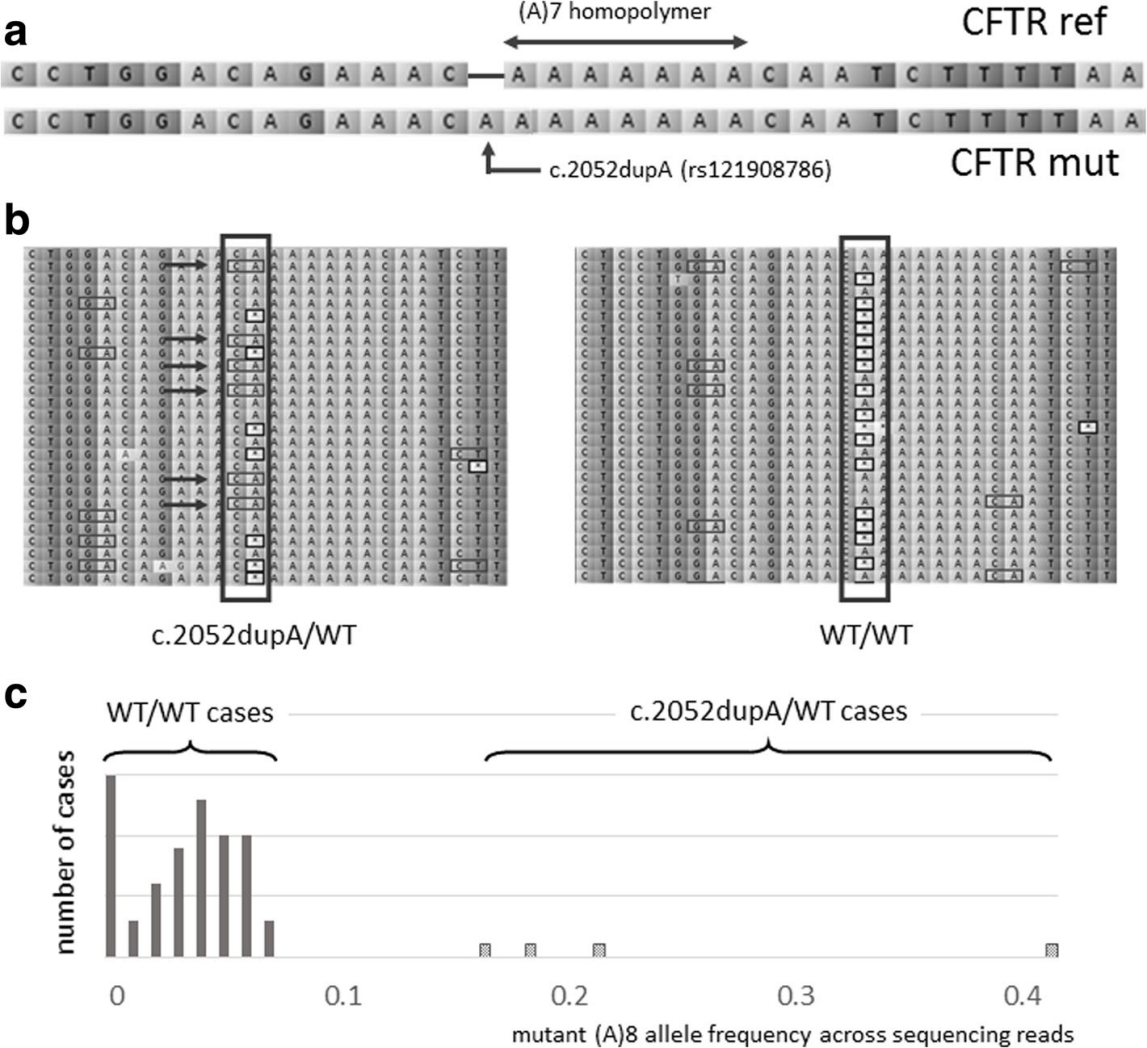
Capability of the semiconductor technology to detect mutations near homopolymer regions. **a**. Mutation c.2052dupA occur in (A)7 homopolymer region of the *CFTR* which is complicated to accurately discriminate with the semiconductor NGS technology **b**. Single sample carrying c.2052dupA mutation demonstrates presence of the variant (A)8 allele in sequencing reads (indicated by arrows) alongside with the (A)7, (A)6 and (A)5 alleles in contrast to sample without mutation, harboring predominantly (A)7, (A)6 and (A)5 alleles **c**. Distribution of (A)8 variant allele frequency across sequencing among all samples. The majority of samples are grouped below 10% allele frequency, while mutation carries have frequencies of 16, 18, 21 and 42%

Although semiconductor sequencing technology is known to be of low accuracy in resolution of mononucleotide repeats [48], we were able to successfully detect all known causative mutations, including single nucleotide deletions and insertions adjacent to these repeats with the use of additional in-house software along and manual sequencing data review. As an example, for the c.2052dupA (rs121908786) mutation, which was detected in four patients, the allele frequencies of the mutant (A)8 sequence were at 16, 18, 21, and 42%, respectively. Across patients without c.2052dupA mutation, these frequencies were limited to maximum of 8%, with peaks in allele frequency distribution at 0 and 4% (Fig. 2). Hence, the discrimination of truly mutant alleles and cases of non-resolution of homopolymeric stretch was achieved. In all four cases, Sanger resequencing confirmed heterozygous nature of these mutations.

**Fig. 3:**
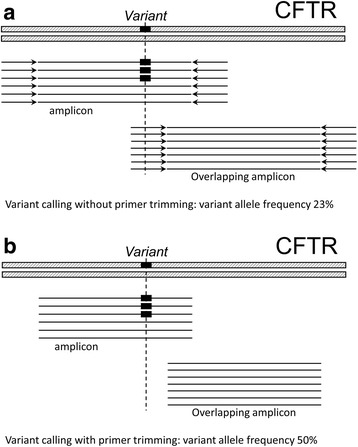
Impact of the primer trimming on variant calling. Detection of the p.Glu92Lys variant was complicated by presence of the technical sequences in data, which lead to false negative calls employing standard pipelines: **a**. Technical sequences (primers) result in reference allele overestimation and influence variant calling **b**. Trimming primers allow to detect mutation in all samples

Overall, our analysis pipeline identified a variety of pathogenic, likely pathogenic, and variants of uncertain significance (VUS), as well as many frequent benign variants (data not shown). Of interest, mutation p.Glu92Lys collocated with a primer hybridization site for one of the pair of overlapping amplicons covering the 4th exon of the *CFTR* (Fig. 3). Thus, overestimation of the wild type allele was observed at pre-trimming stage. After in silico removal of the primer sequences, the mutation was detected successfully. No cases of alleles dropping out due to the hybridization preventing primer binding site variation were seen.

### Identification of disease-causing mutations

Patients homozygous for p.Phe508del were excluded from the analyzed set by pre-screening. Therefore, a study of the greater diversity of genetic variations and correlations between genotype and disease phenotype was enabled this way. In total, this sequencing effort revealed 48 unique mutations, including 17 missense, 7 nonsense, 1 synonymous, 7 splice site, 4 intron variants, 2 inframe, and 8 frameshift deletions in the 84 patients. A mutation was considered a splice site mutation if it was located 4 nucleotides or less from the exon start/end. All mutations were confirmed with direct Sanger sequencing.

Either homozygosity or heterozygosity for the class IV-V mutation were detected in 38 patients out of 84 (45%), while rest of the patients (*n* = 46, 55%) were homozygous for class I-III mutations. Though the majority of patients with the p.[Phe508del];[Phe508del] genotype were excluded after pre-screening, the p.Phe508del allele remained the most abundant, as it was found in 32 patients with a severe phenotype (74%) and in 28 patients with a mild phenotype (68%). Following the homozygous p.Phe508del, the genotype most frequently detected in severe form of CF was p.[Phe508del(;)Ser18_Gly91del], which was seen in four patients (9%). The most frequent genotype identified in mild form of CF was c.[3718-2477C > T(;)1521_1523delCTT], which was seen in 8 patients (22%). Allele c.3718-2477C > T was the second prevalent, with variant frequency of 37% among patients with a mild form of CF, and 16% among entire cohort. In total, we observed 157 pathogenic or likely pathogenic alleles.

We were able to identify at least two causative mutations in 70 patients with NGS alone. For 14 patients, only one mutation was found, and they were subjected to MLPA analysis to identify larger genome variations. Deletion of exons 2-3 and exon 6b-10 duplication were identified in these 14 patients (12 and 2 patients, respectively).

Analysis of the benign polymorphisms revealed heterozygosity for the p.Val470Met detected in 42% of patients and homozygosity - in 46% of patients. Analysis of the IVS8 poly(T) splicing variant revealed the frequency of the 5 T allele at 44%, 7 T allele at 54% and 9 T allele - at 2%. No 12TG or 13TG alleles of the IVS8 poly(TG) splicing variant were identified.

Altogether, a combination of NGS with MLPA was successful in identifying causative mutations in 100% of the CF patients (Table 2). Ten patients out of 84 patients (12%) harbored more than two pathogenic or likely pathogenic mutations or VUS, and, therefore, were classified as having complex alleles (Table 2).

**Table 2:** Causative mutations identified in 84 patients with diagnosed CF

Allele 1*		Allele 2*		n	PT
PANCREATIC SUFFICIENT DISEASE (*N* = 37)					
3849 + 10kbC > T		F508del		8	M
3849 + 10kbC > T		F508del;L467F		1	M
3849 + 10kbC > T;R668C		F508del		1	M
3849 + 10kbC > T;R668C		R1066C		1	M
3849 + 10kbC > T;R668C		2143delT		1	M
3849 + 10kbC > T		I1295Ffs		1	M
3849 + 10kbC > T		S1226*		1	M
3272-16 T > A^†^	R347P^†^		394delTT^†^	1	M
3272-16 T > A		F508del		3	M
2789 + 5G > A		F508del;L467F		1	M
2789 + 5G > A		ex2,3del		1	M
3272-11A > G		F508del		1	M
E92K		E92K		1	M
E92K		F508del		7	M
E92K		ex2,3del		1	M
S1159P		F508del		1	S
R334W		F508del		1	M
R347P		W1282R		1	M
L138ins		F508del		2	M
L1335P		F508del		1	M
I1295Ffs		F508del		1	S
PANCREATIC INSUFFICIENT DISEASE (*N* = 47)					
Y569H		F508del		1	S
G461E		N1303 K		1	S
S1159F		S1159F		1	M
Q98R		2184insA		1	M
Q98R		G542*		1	M
F508del		F508del		6	S
F508del		W1282R		2	S
F508del		S945 L		2	S
F508del		R1070Q;S466*		1	S
F508del		ex2,3del		4	S
F508del;L467F		ex2,3del		1	S
F508del		2143delT		3	S
F508del;L467F		2143delT		1	S
F508del		2184insA		1	S
F508del		N415*		1	S
F508del		R1239=		2	S
F508del		R785*		1	S
F508del		dup6b,10		1	S
F508del		R709*		1	S
F508del		1898 + 1G > C		1	S
F508del		4374 + 1G > A		1	S
F508del		3821delT		1	S
F508del		L15Ffs		1	S
N1303 K		N1303 K		1	S
N1303 K		ex2,3del		1	S
R785*		R1070Q;S466*		1	S
ex2,3del		ex2,3del		1	S
ex2,3del		2184insA		1	S
ex2,3del		N415*		1	S
394delTT		2184insA		1	S
4374 + 1G > T		4374 + 1G > T		1	S
dup6b,10		712-1G > T		1	S
1716 + 1G > A		2043delG		1	S
2118del4		1248 + 1G > A		1	S

Phenotype is displayed in the PT columns (M – “mild”; S – “severe”, where “mild” is defined as homozygous or heterozygous for class IV-V mutations and “severe” as homozygous for class I-III mutations). cDNA variant names according to HGVS nomenclature are given below the table for each variant. Variants were referenced using NM_000492.3. *Identified variants: 3849 + 10kbC > T (c.3718-2477C > T); 3272-16 T > A (c.3140-16 T > A); 2789 + 5G > A (c.2657 + 5G > A); 3272-11A > G (c.3140-11A > G); E92K (c.274G > A); S1159F (c.3476C > T); R334W (c.1000C > T); R347P (c.1040G > C); Q98R (c.293A > G); L138ins (c.412_413insACT); L1335P (c.4004 T > C); Y569H (c.1705 T > C); G461E (c.1382G > A); S1159P (c.3476C > T); F508del (c.1521_1523delCTT); N1303 K (c.3909C > G); W1282R (c.3844 T > C); S945 L (c.2834C > T); R1070Q (c.3209G > A); R1066C (c.3196C > T); ex2,3del (p.Ser18_Gly91del); 2143delT (c.2012delT); 394delTT (c.262_263delTT); 2184insA (c.2052dupA); 1295Ffs (c.3883delA); R1239 = (c.3717G > A);N415* (c.1240_1244delCAAAA); R785* (c.2353C > T); 4374 + 1G > T (c.4242 + 1G > T); R668C (c.2002C > T);dup6b,10 (c.(743 + 1_744-1)_(1584 + 1_1585-1)dup); S466* (c.1397C > A); R709* (c.2125C > T); S1226* (c.3587C > G); 1898 + 1G > C (c.1766 + 1G > C); 1716 + 1G > A (c.1584 + 1G > A); 4374 + 1G > A (c.4242 + 1G > A); 3874-14C > T (c.3874-14C > G); 3821delT (c.3691delT); 2118del4 (c.1984_1987delCTAA); 1248 + 1G > A (c.1116 + 1G > A); L15Ffs (c.43delC); G542* (c.1624G > T); 2043delG (c.1911delG); L467F (c.1399C > T); † Cis/trans position of alleles could not be identified

### Clinical characteristics of patients with different genotypes

Analysis of the clinical expression of cystic fibrosis is usually carried out with respect to the class of mutations detected [49]. From 84 consented patients, we collected clinical information including the pancreatic sufficiency, lung function, and the descriptors of bacterial flora (Table 1). Of note, bias towards higher frequency of pancreatic sufficient CF disease observed in studied patients compared to general CF patient population is possibly caused by the distribution of patient age in our study (range 19-47).

Consistent with previous findings [49], in the cohort of study, the detection of the class I-III mutations in homozygote (*N* = 46) was significantly linked to pancreatic insufficient CF disease (Fisher-test *p*-value < 0.01), while heterozygosity or homozygosity for the class IV-V mutation (*N* = 38) was primarily associated with pancreatic sufficient CF disease. Diabetes cases (*N* = 10) were seen only among patients homozygous for class I-III mutations. For all patients, we retrieved lung function characteristics including FEV and FVC, as well as patient weight and BMI, and found their distributions the same between the two groups, in agreement with previously published results [31]. In both groups, the microbiological profiles of respiratory tract were about the same, with the dominance of Gram-negative flora. Among Gram-positive flora, *Staphylococcus aureus* was observed more frequently in patients with class IV-V mutations (Fisher test *p*-value < 0.01).

### Significantly lower body weight in patients with p.R668C variant present as complex allele c.[2002C > T;3717 + 12191C > T]

Three patients were harboring mutation p.R668C (p.Arg668Cys or c.2002C > T) as part of complex genotypes c.[3717 + 12191C > T(;)1521_1523delCTT(;)2002C > T], c.[3717 + 12191C > T(;)3196C > T(;)2002C > T], and c.[3717 + 12191C > T(;)2012delT(;)2002C > T]. As one can see, in all listed cases this mutation was accompanied by variant c.3717 + 12191C > T (3849 + 10kbC > T). Analysis of CFTR2 database showed that these mutations have already been reported in cis. Mutations c.1521_1523delCTT (p.Phe508del), c.3196C > T (p.Arg1066Cys), and c.2012delT (c.2143delT) are well known as pathogenic. Therefore, it is plausible to pose that in patients analyzed here, the mutations p.R668C (c.2002C > T) and c.3717 + 12191C > T are co-inherited within same chromosome, and that a complex allele c.[2002C > T;3717 + 12191C > T] is fixed in the Russian population.

ClinVar, HGMD classify p.R668C variant as VUS while CFTR2 classify it as non CF-causing. Meanwhile, in silico analysis using all seven algorithms scored this mutation as having a damaging effect on protein function.

Since mutation c.3717 + 12191C > T classifies as a contributor to a mild genotype, all three patients with p.R668C mutation were placed in this group The median FEV for these patients was 64% (28, 75, and 90%) and FVC was 42% (20, 59, and 47%, respectively). No significant differences in the FEV or FVC were seen in these patients in three individual comparisons: i) patients with a mild genotype; ii) patients with c.3717 + 12191C > T mutation; iii) all other patients. Three carriers of p.R668C mutation were also not different from any of these subgroups in their bacterial flora, presence or absence of diabetes and pancreatic sufficiency.

Interestingly, patients with p.R668C mutation had significantly lower body weight (t-test *p*-value < 0.01 for all subgroups). Patients with a c.[2002C > T;3717 + 12191C > T] complex allele had weights of 36, 44, and 48 kg (mean 43; CI 95%, 35-49 kg; mean BMI: 68%), while for all patients, the mean weight was 52 kg (CI 95%, 50-52; mean BMI: 82%), that of patients with a mild genotype was 54 kg (CI 95%, 50-57; BMI: 83%), and that of patients with c.3717 + 12191C > T and no p.R668C allele was 55 kg (CI 95% 48-61; BMI: 81%). Thus, we conclude that p.R668C may modify phenotype and affecting the clinical manifestation of cystic fibrosis, however, the association is based on small sample size and further studies should address the modifying effect of this allele.

### Mutation p.Leu467Phe is found in cis with p.Val470Met and p.Phe508del variants

Mutation p.Leu467Phe (c.1399C > T) was found in four patients. In all cases, this mutation accompanied by pathological p.Phe508del as part of complex genotypes c.[3718-2477C > T];[1521_1523delCTT;1399C > T], c.[2657 + 5G > A];[1521_1523delCTT;1399C > T], c.[54-5940_273 + 10250del21kb];[1521_1523delCTT;1399C > T], and c.[2012delT];[1521_1523delCTT;1399C > T]. Mutations p.Leu467Phe and p.Phe508del are in close proximity to each other, within the 11th exon of *CFTR*. In all four cases, these mutations were overlapped by same set of reads, while another set of reads was mutation-free. Hence, we conclude that in Russian population *cis* position is common for these mutations. Because of that, allele discrimination or other non-sequencing based co-detection of this mutation with p.Phe508del may lead to incorrect diagnosis of CF in the carrier of complex allele, or incorrect ascertainment of genotype in CF patient.

Moreover, within the same haplotype, we identified another common, benign in CFTR2 polymorphism p.Val470Met (rs213950 with MAF 42% based on “1000 Genomes” project). In CF and *CFTR*-related diseases, p.Val470Met was identified as co-segregating with the T5 allele of polymorphic locus Tn [50]. Even though the p.Val470Met has a high allele frequency in the general population and is not associated with CF, co-segregating on the same chromosome with known CF mutation may modify the course of disease [49, 51, 52]. Previous in vitro studies have revealed that p.Val470Met *CFTR* gene products mature more slowly, and their intrinsic chloride channel activity is 1.7 times higher as compared to the wild type protein [51]. Moreover; linkage analysis and case-control studies in CF and non-CF *CFTR*-related disorders have demonstrated that the p.Val470Met locus may affect the overall function of the haploid gene products and alter the penetrance of other CF mutations [50, 52–55].

In our clinical cohort of four patients, we observed no significant alteration in the clinical expression of CF for complex allele described above. In silico predictions of the effect of p.Leu467Phe variant by seven computational techniques had not reached a consensus. Three programs (PolyPhen-2, PROVEAN, Phd-SNP) classified this mutation as pathogenic, while in case of all confirmed CF-causing mutations, pathogenicity of mutation was asserted by at least five out of seven algorithms.

### Mutation p.Arg1070Gln in cis with p.Ser466* is associated with mild pulmonary obstructions

In two patients with p.Arg1070Gln (c.3209G > A) other two pathogenic variants were detected, c.[1521_1523delCTT(;)1397C > A(;)3209G > A] and c.[2353C > T(;)1397C > A(;)3209G > A]. Co-location of these variants p.Arg1070Gln and p.Ser466* within the same chromosome was reported previously in association with pancreatic-insufficient CF [27]. In this study, clinical characteristics of patients with p.Arg1070Gln were consistent with previous observations. Both patients had pancreatic insufficient CF disease, though larger FVC (107 and 109%) and FEV (90 and 111%) as compared to all other patients (mean FVC of 67% (Ci 95%, 63-72%); mean FEV of 47% (CI 95%, 37-48%) were observed. Increases in FVC and FEV were also observed in comparisons to patients with severe phenotype. No statistically significant differences were seen for any other clinical characteristic.

## Discussion

In this study, we performed a comprehensive analysis of the variants in the *CFTR* gene by targeted semiconductor NGS in a cohort of 84 CF patients prescreened for 30 most common *CFTR* mutations. Exclusion of a majority of p.Phe508del homozygotes allowed us to explore wider range of rare mutations and, thus, contribute to determining the mutation spectrum in Russian patients (Table 2, Fig.1c).

Previous reports on outcomes of genetic tests performed in various regions of Russia indicate that conventional methods allow detection of a pair of causative mutations in 63.8% of patients with established diagnosis of CF [56]. In this study, we combined the detection of point mutations as well as short insertions and deletions by NGS with MPLA for CNV, and achieved identification of a pair of disease-causing variants in all patients analyzed. Therefore, our study points that combination of NGS with MPLA outperforms orthogonal screening panels in populations of mixed origin.

In Russia, region-specific spectra of the *CFTR* mutations were previously reported [57, 58]. As a majority of patients enrolled in present study had originated in central regions of Russia, observed mutation spectrum was majorly consistent with that collected by nationwide register of the CF patients [56]. In our study cohort, an enrichment with low prevalent mutations was achieved by excluding the patients homozygous for p.Phe508del at pre-screening. Nevertheless, the most prevalent mutations identified in our study matched the list of most prevalent CF mutations nationwide: c.3718-2477C > T (nationwide prevalence of 2.18%), p.Ser18_Gly91del (5.94%), c.274G > A (2.58%), c.2012delT (1.67%), c.3909C > G (1.46%), c.2052dupA (1.69%). These mutations are listed in descending order of frequency in our study.

In contrast to the mutation panels, NGS technology is comprehensive, as it allows analysis of variations within the whole target sequence. Because of this advantage, the detection of mutations with presumed modifier properties is possible. In present study, ~ 10% of the patients with CF were shown to harbor more than two *CFTR* variants, which might be either disease-causing or modifying. In particular, p.Arg1070Gln and p.Leu467Phe recurrently occurred together with known causative mutations, thus, most likely serving as disease modifiers.

We have also noted that, at least in one instance, the complexity of haplotype may lead to erroneous diagnosis during newborn or neonatal screening. A case of this kind had been described previously in Polish population: clinical evaluation of a patient with genotype p.[Leu467Phe;Phe508del] did not confirm the presence of CF [59]. Comparing this case with four cases where these two mutations were present in cis, we could see that misdiagnosing a carrier for a cystic fibrosis patient may be a real problem.

For all two mutations with modifier potential, p.Arg1070Gln and p.Leu467Phe, an analysis of annotations was performed, and relatively low confidence of annotation was noted. For example, in CTFR database p.Arg1070Gln is defined as VUS. Therefore, further inquiry into the significance of these variants was undertaken with an aid of computational biology.

There are numerous computational tools which predict the variant’ effect on protein function [60, 61]. Although the majority of them are intended for the binary classification into pathogenic or benign mutations, the judgement is a score-based. These score are shown to correlate with residual activity of mutated protein. Therefore, these tools may be useful for predicting the expressivity of pathogenic mutations [62]. Since CF is a disease with varying severity, we suggest that incorporating several mutation effect prediction tools and a continuous output score instead of binary prediction, may improve clinical judgments about the degree of severity of the disease.

**Fig. 4:**
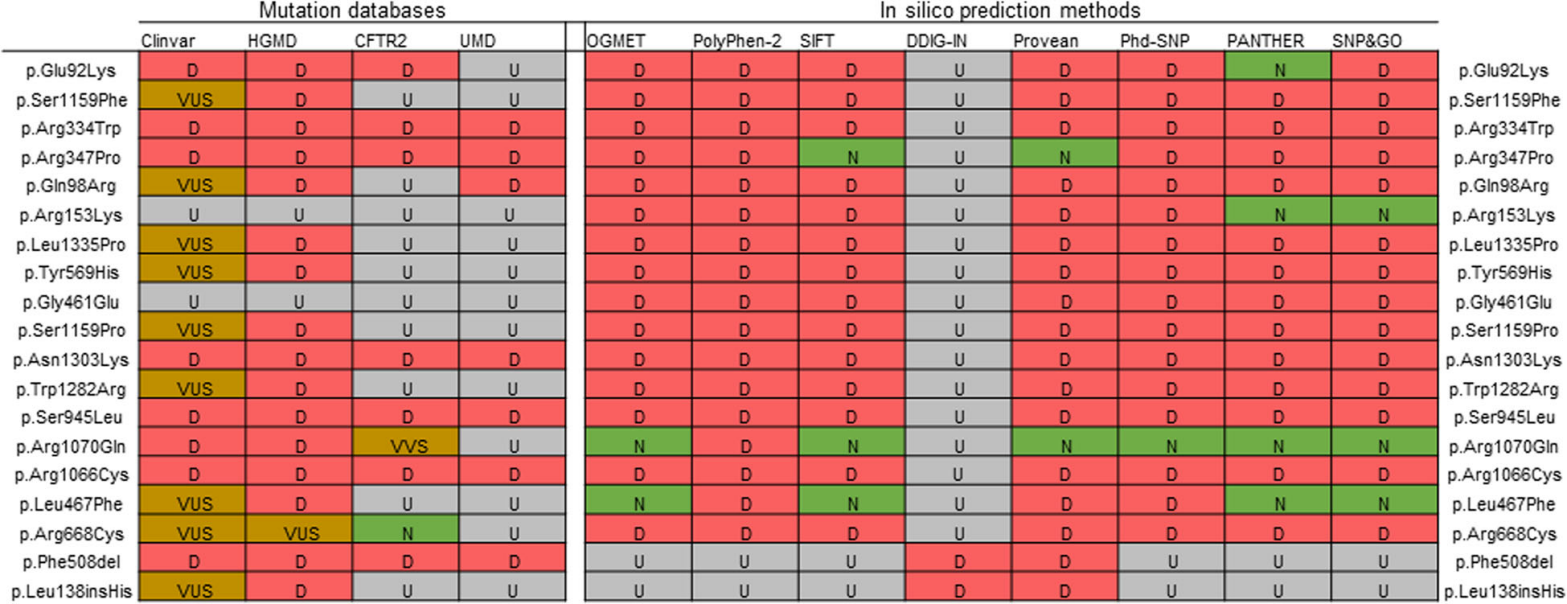
Missense substitutions and in-frame deletions/insertions annotated in accordance with used variant databases and computational tools. VUS stands for Variant of Uncertain Significance; D – pathogenic; N – benign/non CF-causing; U – annotation could not be assessed; VVS – variant of varying clinical significance according to CFRT2 variant classification

Considering the multiple complex genotypes, we assigned 14 out of 17 detected unique missense mutations to the category of disease causative. The other two, p.Arg1070Gln and p.Leu467Phe, were seen only in combinations with other pathogenic mutations, as part of complex genotypes. For them the predictions were highly diverse, indicating that, even if these mutations influence the function of *CFTR* gene product, this influence is lower than that of disease-causing ones. However, p.Arg1070Gln mutation is located in *cis* with the upstream p.Ser466* nonsense mutation, therefore, in this particular case its modifying effects cannot manifest. By four computational assessment techniques (Fig. 4), variant p.Leu467Phe was classified as benign methods; our analysis of patients’ phenotypes did not show any correlations. Therefore, in silico predictions for the presumably modifying mutations were consistent with the patient’s disease characteristics. Combined with the fact that predictions for the 14 presumably disease-causing mutations were in concordance across the majority of tools, this demonstrates a value of proposed consensus approach for interpreting novel mutations or variants of uncertain significance uncovered during prenatal or newborn screening.

While computational tools may aid in initial annotation of modifying mutations, clinical significance of such variants has to be confirmed by comparative analysis of patient’s phenotypes. Meanwhile, increasing an adoption of NGS into the routine of CF diagnostics may facilitate accumulation of data describing complex genotypes, including these with the variants of varying significance. Indeed, an analysis of presented cohort showed that complex allele p.[Arg1070Gln, Ser466*] is linked to milder form of pulmonary obstructions.

It is important to note that despite high diagnostic yield, NGS technology requires substantial laboratory and analytic efforts, and is relatively difficult to standardize. For instance, in this study, the standard bioinformatics pipelines were inefficient for analysis of cohort. In 9 patients, p.Glu92Lys mutations were detected at a primer hybridization site at one of overlapping amplicons covering the 4th exon of the *CFTR* (Fig. 3). This position of the mutation led to an excess of reference nucleotides at the mutation site, which is not assumed by the variant caller’s statistical models and, therefore, cause a strand bias misdirecting towards a sequencing error. As a consequence, both SamTools and GATK, two the most commonly used variant callers, repeatedly miss this mutation, when used under standard parameters. Identification of the mutation is possible only after the trimming of primers, performed by in-house software (see Additional file 1). We should note that both Trimmomatic [63] and Cutadapt [64], which are capable of removing “technical” sequences from fastq files, are not specific for the task. Software mentioned above removes both adaptors and primers, and because of that, work at pre-alignment stage of the data processing. As primers are parts of the genome, they anchor alignments, and, therefore, are aiding in detecting small deletions and insertions, especially near ends of the reads. Hence, primer trimming should be performed after the alignment, and require special tool. Following primer trimming, p.Glu92Lys mutation was successfully identified with both variant callers in all the samples.

Furthermore, in our samples, we were able to detect mutations located near repetitive DNA sequences, including small deletions and insertions, though the development of in-house scripts was required. The coding sequence of the *CFTR* possesses several homopolymer regions, including the (A)7 homopolymer tract with c.2052dupA. Previous papers showed that semiconductor NGS protocols may generate false negative [65] or false positive [22] calls at this site. Using quantitative discrimination threshold, we were able to detect c.2052dupA mutation along with the other similar mutations. Nevertheless, large variation of the coverage across patients did not allow us to detect CNV, including exons 2,3 deletion and 6-10 duplication, though several previous studies have demonstrated that detection of this type of mutation with targeted NGS protocols may be achieved [22, 66].

## Conclusion

We have demonstrated that a combination of Ion Torrent PGM sequencing with MLPA outperforms conventional methods in its diagnostic yield allowing to *i)* improve the quality of the molecular analysis in CF in terms of detecting causative mutations *ii)* identify modifying variants within complex genotypes.

Nevertheless, our study demonstrates that the choice of bioinformatics pipeline plays a crucial role in detecting clinically significant variants and highly influences diagnostic yield. Moreover, use of software from the sequencing platform supplier may not be sufficient. Our study supports the view that validation of NGS system, including its analytical sensitivity and specificity should be performed for variant separately, and any changes in chemistry, enrichment protocols, or the data analysis require re-validation of the whole test system [66].

## Additional file

Additional file 1: Supplementary methods. Primer trimming during NGS data pre-processing. (DOCX 18 kb)

## Abbreviations

CF: Cystic Fibrosis
NGS: Next Generation Sequencing
VUS: Variants of Uncertain Significance
