# Supplementary material for: Targeted sequencing reveals complex, phenotype-correlated genotypes in cystic fibrosis
Source: BMC Med Genomics. 2018 Feb 13;11(Suppl 1):13. doi: 10.1186/s12920-018-0328-z (PMC5836842; doi:10.1186/s12920-018-0328-z)
Supplement: Supplementary file 1 — Supplementary methods. Primer trimming during NGS data pre-processing. (DOCX 18 kb) [file 12920_2018_328_MOESM1_ESM.docx]

**Primer trimming during NGS data pre-processing**

PCR amplification remains the most convenient method of target DNA enrichment during NGS resequencing protocols when the size of target region is order of ten to hundred kilobases. In this case primers used for DNA amplification represent technical sequences of the synthetic origin, whereas insert sequences located between two primers constitute the major part of amplicons and represent analyzing objects. Insert along with primers other technical sequences also named amplicon. Considering the fixed sequence of primers, preserving them in sequencing data may influence allele prevalence in sites of two or more amplicons intersection. As result technical sequences contamination in reads pile-ups, false-negative variant calls may occur in such regions. Therefore, primer trimming should be performed before variant calling in order to improve the quality of the analysis. Considering that primers, in contrast to other technical sequences, represent genome-derived sequences, they can successfully be mapped along with the inserts. Practice shows that aligning inserts with primers together significantly improves the quality of alignment, especially in case of the insert terminating small deletions/insertions. Consequently, primers trimming is reasonably to perform after alignment stage.

Prettry performs amplicon trimming and suits best for NGS target resequencing protocols employing PCR based target enrichment. Given the sequences of inserts along with input FASTQ or SAM files, Prettry performs searching of the technical sequences non-relevant to the inserts in each read and remove it. Considering that the main goal is to remove primer sequences, it is recommended to run Prettry on SAM files after alignment.

Prettry workflow consist of two main stages - associating read with one or more target insert and directly trimming. Associating read with one or more target insert sequences starts with decomposing DNA into overlapping *k-*mers (*i.e.* nucleotide sequences of length *k*). Given a positive integer value *k*, each of the 4*^k^*, *k*-mers can be stored with only *n_k_* bits by using an *n-*bit binary code *b_n_*. Binary coding b_2_ is defined as b_2_(A) = 00, b_2_(C) = 01, b_2_(G) = 10, b_2_(T) = 11. As a result, each *k*-mer is bijectively associated to the unique binary number of 2*k* bits. Usual processors support 64 bit long words, so binary coding b_2_ allows *k*-mers with *k* ≤ 32 to be easily computed and stored. Given an insert sequence, the binary representation of its k-mers is computed employing three basic bits operations: bitwise OR (|), bitwise AND (&), and bit left shifting ($\ll$). More formally, if *w_i_*, is the binary representation of the *k*-mer starting at position *i* and ending at position *i+k-1*, the binary representation *w_i+1_* of the next *k*-mer is *w_i+1_*=((*w_i_* $\ll$ *n*) | *b_n_*(*s_i+k_*))&*mask_n_* , where *s_i+k_* is the character state at position *i+k*, and *mask_n_* is a constant binary representation of *n^k^-1* that allows setting to zero all bits shifted beyond the *n^k^*-th one. Following this approach, the list of every *k*-mer from a nucleotide sequence *l* is computed at time *O(l)*.

Given a fixed integer value k (13 by default), *k*-mer decomposition performed for every insert sequence and respective reverse complement. Hash-table *D* is further built by associating every *k*-mer binary representation with a list that contain 1-based insert numbers.

For every read with length *l*, three groups of positions are defined as $p\left( l \right)=\left( \left\{ \frac{l}{2}-\frac{k}{2}, k, l-2k \right\}, \left\{ \frac{l}{3}-\frac{k}{2},\frac{2l}{3}-\frac{k}{2} \right\}, \left\{ 0, l-k \right\} \right)$. Given a single-end read sequence of length *l*, binary representation of k-mers starting at the m-th group positions can be computed, increasing *m* until the corresponding key is found in hash-table *D*. Respective hash-table values are further concatenated in order to form a list of potential inserts for the read. In case of paired-end or mate pair reads with length *l_1_* and *l_2_*, potential insert sets A_1,[1:m]_ and A_2,[1:m]_ are obtained by uniting values, corresponding in the hash-table *D* to *k*-mers binary representation at positions from the first *m* groups in *p(l_1_)* and *p(l_2_)* respectively until potential amplicons *A = A_1,[1:m]_*$\cap$*A_2,[1:m]_* is no longer empty. If *A* remains empty after *m = 3, A = A_1,[1:3]_*$\cup$*A_2,[1:3]_* is used.

At trimming stage all single-end reads are aligned with every previously associated insert sequence employing Smith-Waterman local alignment. The read is trimmed according to the best alignment among the potential inserts if the score *s* is at least *t*$\times$*l* where *t* is predefined threshold (1/2 by default) and *l* is the length of the read. If *s ≥ t*$\times$*l* and the best local alignment took place between the read positions *j_1_* and *j_2_* than the read is trimmed to its subsequence starting at positions *j_1_* and ending at position *j_2_*. The read remains untrimmed if *s < t*$\times$*l*. Paired-end and mate-pair reads are aligned to insert sequences and respectively trimmed at once.

Prettry is available for download at https://github.com/tprodanov/Prettry
